# Leveraging Oral Drug Development to a Next Level: Impact of the IMI-Funded OrBiTo Project on Patient Healthcare

**DOI:** 10.3389/fmed.2021.480706

**Published:** 2021-03-05

**Authors:** Bart Hens, Patrick Augustijns, Hans Lennernäs, Mark McAllister, Bertil Abrahamsson

**Affiliations:** ^1^Drug Delivery and Disposition, Department of Pharmaceutical and Pharmacological Sciences, KU Leuven, Leuven, Belgium; ^2^Drug Product Design, Pfizer, Sandwich, United Kingdom; ^3^Department of Pharmaceutical Biosciences and Technology, Uppsala University, Uppsala, Sweden; ^4^Oral Product Development, Pharmaceutical Technology and Development, Operations, AstraZeneca Gothenburg, Mölndal, Sweden

**Keywords:** IMI, EFPIA, oral biopharmaceutical tools, pharmacokinetic, oral absorption, patient health care, oral formulations

## Abstract

A thorough understanding of the behavior of drug formulations in the human gastrointestinal (GI) tract is essential when working in the field of oral drug development in a pharmaceutical company. For orally administered drug products, various GI processes, including disintegration of the drug formulation, drugrelease, dissolution, precipitation, degradation, dosage form transit and permeation, dictate absorption into the systemic circulation. These processes are not always fully captured in predictive *in vitro* and *in silico* tools, as commonly applied in the pre-clinical stage of formulation drug development. A collaborative initiative focused on the science of oral biopharmaceutics was established in 2012 between academic institutions and industrial companies to innovate, optimize and validate these *in vitro* and *in silico* biopharmaceutical tools. From that perspective, the predictive power of these models can be revised and, if necessary, optimized to improve the accuracy toward predictions of the *in vivo* performance of orally administered drug products in patients. The IMI/EFPIA-funded “Oral Bioavailability Tools (OrBiTo)” project aimed to improve our fundamental understanding of the GI absorption process. The gathered information was integrated into the development of new (or already existing) laboratory tests and computer-based methods in order to deliver more accurate predictions of drug product behavior in a real-life setting. These methods were validated with the use of industrial data. Crucially, the ultimate goal of the project was to set up a scientific framework (i.e., decision trees) to guide the use of these new tools in drug development. The project aimed to facilitate and accelerate the formulation development process and to significantly reduce the need for animal experiments in this area as well as for human clinical studies in the future. With respect to the positive outcome for patients, high-quality oral medicines will be developed where the required dose is well-calculated and consistently provides an optimal clinical effect. In a first step, this manuscript summarizes the setup of the project and how data were collected across the different work packages. In a second step, case studies of how this project contributed to improved knowledge of oral drug delivery which can be used to develop improved products for patients will be illustrated.

## Introduction

In October 2012, the IMI/EFPIA-funded initiative “Oral Bioavailability Tools (OrBiTo)” project was launched and established a 5 year ongoing collaboration (extended with one extra year) between academic institutions and pharmaceutical companies with one major goal: refining and accelerating the formulation drug development process in order to serve better drug products to patients. A total of 13 pharmaceutical companies, 9 universities, research organizations, public bodies, one regulatory body, one non-profit research organization and three small/medium enterprises (SMEs) participated in this project (1). The project was initiated by defining the state-of-art knowledge at the start of each work package (2–4). As traditional formulation development typically relies on an empirical approach requiring testing to determine the impact of changes during the development cycle, this project aimed to create a more rational and scientific framework to assist formulation scientists in developing better formulations customized to the patient's needs. The mission statement of the project was: “Through partnership, collaboration and data sharing, we will develop our fundamental knowledge of the gastrointestinal (GI) environment to deliver innovative biopharmaceutics tools which will accurately predict product performance over a range of clinically relevant conditions. The integration of *in vitro* and *in silico* approaches will provide a biopharmaceutics toolkit, validated using clinical data, to accelerate drug development.” After oral administration, a drug product will have different transit times throughout multiple regions in the GI tract. In order to understand how much drug will reach the systemic circulation and which plasma-concentration profile is achieved, it is of utmost importance to understand the anatomy and ongoing physiology that regulate the householding of the human GI tract. By gathering in-depth knowledge about how the GI tract functions, scientists will better understand how differences in systemic plasma concentration-time profiles may occur between and within patients after intake of an approved oral drug product. This has previously been achieved in specially designed oral formulations for patients with Addison's and/or Parkinson's disease (5–8).

After intake, the dosage form needs to disintegrate in order to release the drug compound. Subsequently, the drug needs to dissolve in order to generate a driving force for intestinal absorption. Only then, can drug molecules permeate through the intestinal wall, reach the systemic circulation, and find their way to the site of action. Depending on the physicochemical characteristics of the compound and the formulation properties, some physiological processes (e.g., motility, gastric emptying, pH, biliary secretion) can become critical parameters for dictating drug absorption into the systemic circulation. When implementing these physiological variables into predictive *in vitro* and *in silico* computational biopharmaceutical tools, predictions can be made taking into account the most extreme situations (i.e., in terms of minimal and maximal systemic exposure of the drug) toward a patient population. Defining these physiological GI variables and using them as covariates in *in silico* simulation tools together with population based pharmacokinetic-pharmacodynamic modeling and quantitative systemic pharmacological predictions, it becomes a useful approach to predict the optimal dose for each patient suffering from a chronic/acute disease (i.e., personalized medicine) (9).

Within “OrBiTo,” five discrete work packages (WP) were constructedto assure the successful completion of all pre-defined goals. Based on the outcomes from each work package, an intensive data analysis was performed and a decision tree was developed for different kinds of formulations and compounds of interest (e.g., immediate-release formulation for a weakly basic compound). The decision tree will assist formulation scientists in selecting the most appropriate *in vitro* dissolution test (eventually coupled with computational modeling) to better understand what can be expected from this formulation (in terms of systemic exposure) if it would be given to patients in the later stage of drug development (10). The decision tree can be downloaded from the following website: www.orbito-dissolution.eu. In the following paragraphs, a more detailed description of each work package will be discussed including the pre-defined goals. In addition, two specific case studies of how this project could contribute to improved patient healthcare will be demonstrated for an optimized drug formulation of the poorly soluble compound posaconazole (i.e., antifungal agent) and a second example will demonstrate how different intake conditions (sparkling vs. still water) were tested for paracetamol (i.e., pain-reliever) and resulted in unexpected differences with respect to the systemic concentrations.

## Organization of the Different Work Packages Throughout the “ORBITO” Project

**Figure 1:**
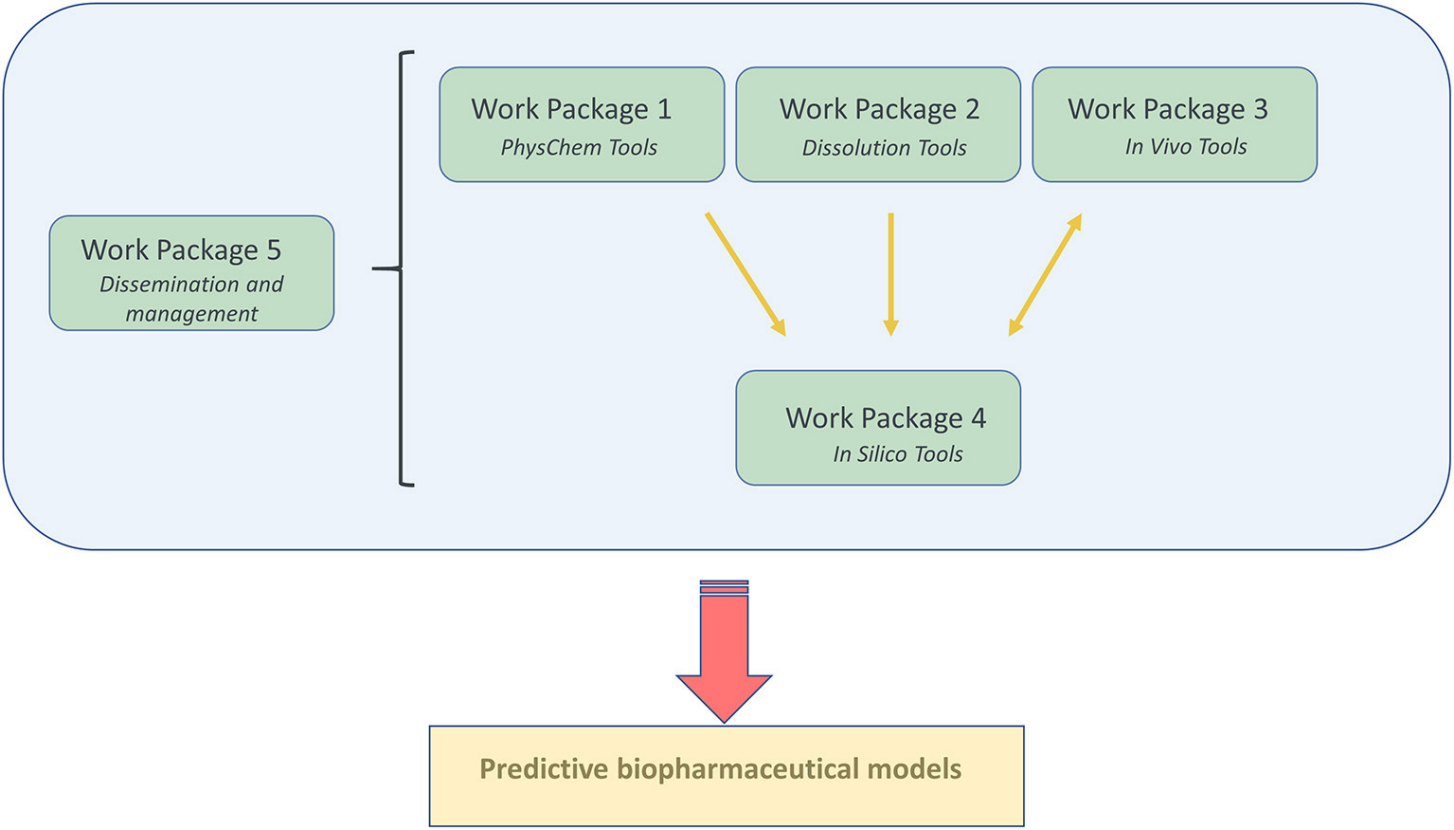
A graphical illustration of the organization of the different work packages (WP) and how they are closely linked to each other.

To reach for the ultimate goal which is “Transform our ability to predict the *in vivo* performance of oral drug products across all stages of drug development,” a well-organized project plan was drafted to guide the consortium effort toward reaching the desired target. The “OrBiTo” project was divided into five different work packages (WPs), with each work package focusing on a specific theme (Figure 1).

Every single WP has its specific kind of expertise and coordinated by two WP leaders; one academic and one industry leader.

## WP 1—*In Vitro* Physchem Tools

*WP Leaders: Rene Holm, Janssen Pharmaceutica (initially joined the project affiliated with the Lundbeck company) and Anette Müllertz, Copenhagen University*.

WP 1 focuses on the physicochemical properties of the active pharmaceutical ingredient (e.g., molecular weight, pKa, LogP, etc.) to find a clear link between these properties and biopharmaceutical characteristics of the compounds (e.g., solubility, dissolution kinetics and/or permeability). In order to significantly contribute to the understanding of molecular biopharmaceutics, WP1 has two cornerstones:

1) A structurally diverse set of active pharmaceutical ingredients (APIs) with the focus on poorly soluble compounds (BCS class II and IV), selected primarily among the EFPIA API to cover a representative chemical range (in terms of pKa values, molecular weight, etc.). To this space, various BCS class I and III are added characterized by a high solubility and high (BCS class I) or low (BCS class III) permeability.
2) A set of simulated gastrointestinal media (SGIM), reflecting compositions of the human GI fluids in the fasted and the fed state.

**Figure 2:**
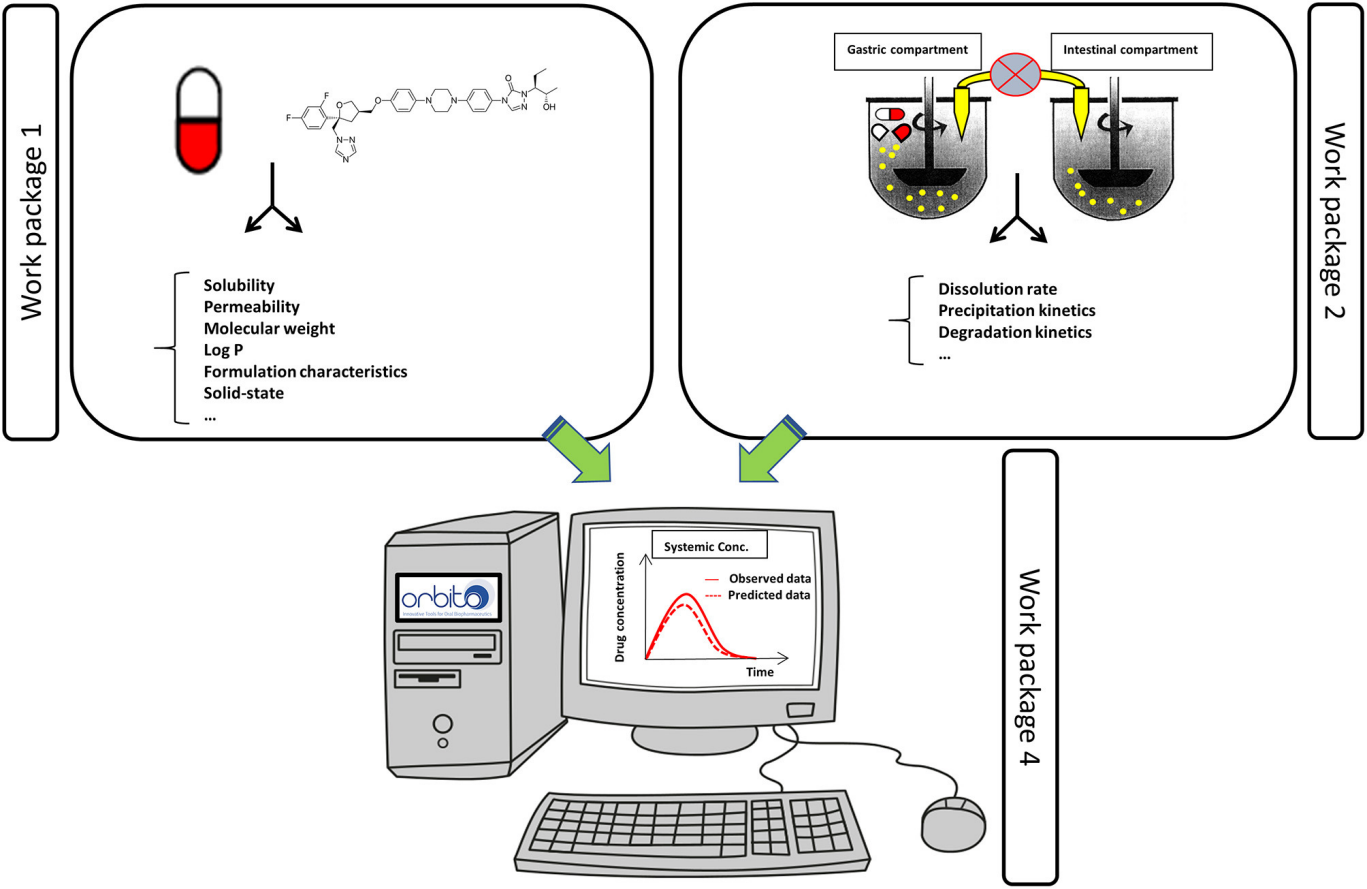
Data obtained from WP 1 and 2 were used as an input for the *in silico* computational tools as described in WP 4 to predict the systemic exposure of the drug. Observed systemic data were obtained from data collected from WP 3.

The sets of API and SGIM were the basis for the standardized, validated physicochemical tools that are developed in WP 1. These models will improve the current physicochemical profiling of APIs, by comparing and linking to *in vivo* data, and thereby securing relevance for API *in vivo* solubility and permeability. Novel methodologies for dissolution rate, supersaturating propensity including re-crystallization/precipitation, intestinal permeability including the impact of mucus diffusion, and surface activity profiling, were developed as described below. In addition, new *in silico* tools for predictions of biorelevant physicochemical variables making use of the experimental data were devised. Specific attention was given to compounds suffering from a low aqueous solubility which are categorized as BCS class 2 and 4 compounds according to the Biopharmaceutics Classification System (BCS) (11). Most of the drug molecules that are populating the pharmaceutical pipelines are drug molecules characterized by a low aqueous solubility (12). This WP is closely linked with WP 2 (“*In vitro* tools—understanding the formulation”) using the knowledge and results obtained from the physicochemical studies and models as listed in WP 1. Moreover, the obtained data from WP 1 served as physicochemical parameter inputs for integrated modeling and predictive tools developed in WP 4. These computational *in silico* models were depending on input data such as solubility, permeability and dissolution values, which are generated by WP 1 and WP 2 (Figure 2).

## WP 2—Dissolution Tools

*WP Leaders: James Butler, GlaxoSmithKline Research and Development Ltd. and Patrick Augustijns, KU Leuven*.

**Figure 3:**
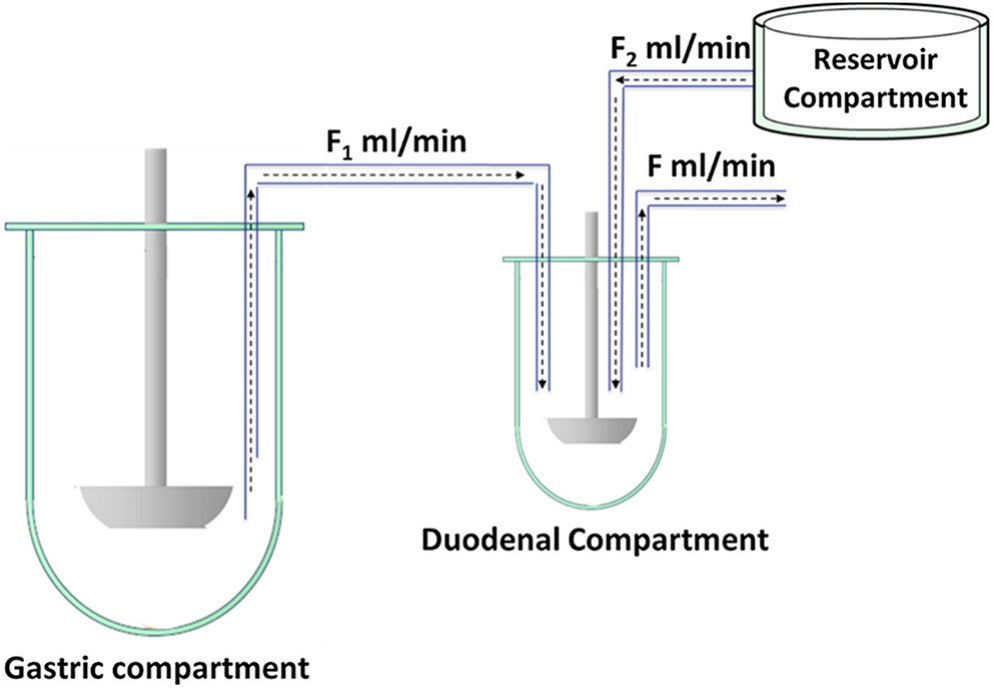
Schematic representation of BioGIT. F_1_ and F_2_ are the incoming flow rates and F is the outgoing flow rate; F = F_1_ + F_2_. Figure adopted from Kourentas et al. with permission. Copyright Elsevier 2016 (13).

The main objectives of WP 2 were (i) to establish biorelevant *in vitro* tools that are able to predict the *in vivo* performance of drug formulations and (ii) to develop a decision tree afterwards that can assist formulation scientists/developers in the selection of the most suitable *in vitro* dissolution test that will give the most predictive outcome for their compound/formulation of interest. The dissolution models that were subject of interest were optimized and validated to improve their predictive accuracy. To do so, *in vivo* data derived from WP 3 served as a reference to optimize/validate these current models. An example of a well-established *in vitro* model and that was validated during the “OrBiTo” project is the Biorelevant Gastrointestinal Transfer (BioGIT) model, representing the upper part of the GI tract consisting of a gastric and a duodenal chamber (13, 14). An extra vessel is added to the model to simulate the intestinal secretions (Figure 3).

An overview of other *in vitro* models that were optimized and/or validated during the “OrBiTo” project, can be found elsewhere (15). The optimization of frequently applied *in vitro* models will clarify the impact on clinical pharmacokinetic (PK) studies to explore the link between formulation design and performance. To asses the likely systemic exposure of a drug after oral intake by a patient, the obtained data from WP 1 and 2 can both be used as an input for computational tools (as described under WP 4) to reproduce the simulated plasma concentration-time profiles.

## WP 3—*In Vivo* Tools

*WP Leaders: Marcus Brewster, Janssen Pharmaceutica—Achiel Van Peer, Janssen Pharmaceutica—Patricia Zane, Sanofi—Peter Langguth, Johannes Gutenberg Universität Mainz*.

To fully understand the intraluminal behavior of a drug in the GI tract, a plethora of *in vivo* techniques were applied during the “OrBiTo” project (16). For example, the clinical aspiration technique has become a gold standard to directly measure drug concentrations along the human GI tract (i.e., stomach, duodenum, and jejunum) (17). After oral administration of a drug, GI fluids can be aspirated as a function of time and, in parallel, blood samples can be collected to investigate the plasma pharmacokinetics of the drug. The aspirated fluids and plasma samples can be analyzed for drug content and a *post-hoc* analysis can be performed to observe a potential link between the intraluminal concentration-time profiles and the deconvoluted plasma exposure, reflecting the intestinal absorption rate and extent of the drug. From that point of view, inter- and intrasubject variability can be explained and the impact of GI physiology (e.g., pH) on the obtained concentrations may be identified (18, 19). In a study by Rubbens and co-workers, concentrations of indinavir were measured after oral administration of one capsule under different test conditions: (i) fasted state, (ii) fed state and (iii) fasted state with concomitant proton pump inhibitor (PPI) use. The results of this study demonstrated that the reported reduction in indinavir's bioavailability after concomitant PPI administration was caused by an elevated gastric pH resulting in less indinavir in solution in the human GI tract. A negative food effect was observed in the fed state conditions due to the intestinal micellar entrapment of indinavir (20).

Besides intraluminal drug concentration profiling of the drug, magnetic resonance imaging (MRI) was used as a robust tool to assess (i) the volume of GI fluids present in the human GI tract and (ii) the gastric emptying and intestinal transit times of specific dosage forms (when magnetically labeled) (21, 22). The measured volumes along the GI tract are extremely valuable as these fluids are necessary for a drug to dissolve; a drug needs to dissolve in the intestinal fluids before being eligible for intestinal permeation (23). MRI is a valuable tool to graphically illustrate the dynamic movement of the fluids and dosage form throughout the GI tract (24). During the “OrBiTo” project, an MRI study performed by Grimm et al. demonstrated the fast gastric emptying of water in fed state conditions (22). Different types of meals were given prior to water administration and it was observed that the gastric emptying of water under fed state clinical trial conditions is as fast as under fasted state conditions. It was seen that water could easily be removed from the stomach using a so-called “stomach road” or “*magenstrasse*.” The texture of solid meals and a higher amount of solid food components turned out to favor the presence of the “*magenstrasse*” (25).

## WP 4—*In Silico* Tools

*WP Leaders: Xavier Pepin, AstraZeneca (initially joined the project affiliated with the Sanofi company) and Amin Rostami, University of Manchester*.

In essence, the objectives proposed within WP 4 are closely linked with the outputs from WP 1-3. The *in silico* computational models which were the focus of WP4 can be “fed” with the gathered data from WP 1 (*in vitro* PhysChem tools) and WP 2 (dissolution tools) and the predicted outcomes (e.g., plasma concentration-time profiles) can be compared with the observed systemic exposure data, as measured during the clinical studies in WP 3. By iterative approaches and the testing of several inputs to the models coupled to model refinements, the ultimate aim is to improve the accuracy and precision of bioavailability predictions. Commercially available software tools (e.g., Simcyp®, GastroPlus™) are helpful to assist pharmaceutical companies in an early stage of drug development to address the “druggability” of a new API to become a marketed drug product, that will generate sufficient therapeutic concentrations in patients after oral administration of the drug product (2). These models take into account the physiological functions of human organs represented by mathematical algorithms. Based on these equations with API and formulation specific data provided by physicochemical and biopharmaceutical characteristics, a mass transport analysis of the drug throughout the different compartments of the human body can be established, indicating how much drug will appear in the different organs after oral administration of the drug to humans. For both GastroPlus™ and Simcyp®, a workspace was created that could predict the intraluminal and systemic concentrations of posaconazole, a weak base, that was administered to healthy subjects at different doses and as different formulations (26, 27). The validation of these workspaces was conducted by directly comparing the observed intraluminal and systemic data, obtained from a clinical aspiration study that was performed in WP 3, with the predicted intraluminal and systemic data (see below).

## WP 5—Management Activities and Dissemination

*Scientific coordinators and managing entity: Bertil Abrahamsson & Martin Berntsson, AstraZeneca—Peter Langguth, Johannes Gutenberg Universität Mainz—Mark McAllister, Pfizer—Hans Lennernäs, Uppsala University—Erik Sjögren, Uppsala University—Christer Karlsson, AstraZeneca—Jenny Ottosson, AstraZeneca*.

The managing team was responsible to assure that all deliverables were accomplished at the pre-defined times. Monthly conference calls and annual face-to-face meetings were indispensable to keep up with the process of this ambitious project. With the extra help of all Ph.D. students and postdoctoral researchers (united as “Young OrBiTo”), numerous initiatives and activities (e.g., webinars, poster sessions and a workshop) were established to share our generated results with a broader audience. Prior to sharing the generated results with the public, all data were first disseminated on the online platform “Sharepoint,” giving the chance to all collaborators to give their personal comments/remarks before the data will be publicly distributed.

## The Impact of the Orbito Project on Patient Healthcare: Case Examples With Posaconazole (Noxafil®) and Paracetamol (Dafalgan®)

### An Optimized Formulation Strategy for Posaconazole to Improve the Oral Bioavailability in Patients

Posaconazole (weak base; pKa 3.6 and 4.6) is used for prophylaxis for invasive fungal infections (IFIs) among patients who are at high risk of developing these infections (e.g., immunocompromised patients). Posaconazole is commercially available as Noxafil® suspension (40 mg/mL) and, more recently, as Noxafil® delayed-release tablet (100 mg). Both formulations are manufactured by MSD Research laboratories (Merck Sharp & Dohme Corp., Kenilworth, NJ, USA). The variable PK of posaconazole when using the oral suspension formulation may limit the therapeutic response in some patients. Posaconazole shows a positive food effect (i.e., increased systemic exposure) (28). It was hypothesized that the intake of food increases the solubility of posaconazole in the intestinal environment and, therefore, increase the driving force for intestinal absorption, resulting in a higher systemic exposure compared to fasted state conditions. Another study revealed a high intersubject variability in systemic exposure, especially when the suspension was taken under hypochlorhydric conditions [e.g., concomitant intake of a proton-pump inhibitor (PPI)] by patients/healthy subjects. A mechanistic study by Walravens et al. demonstrated how the concomitant intake of a PPI resulted in less gastric dissolution of posaconazole (due to the elevated gastric pH), and, subsequently, extremely low intestinal concentrations after GI transfer (29). The concomitant intake of posaconazole with a can of Coca-Cola® resulted in an increase in systemic exposure compared to the test condition (i.e., intake ofthe suspension with 330 mL of water). The authors suggested a prolonged gastric residence time in combination with a more acidic environment in the stomach compared to fasted conditions (pH 2) as a beneficial strategy to generate increased gastric concentrations, which, upon transfer, will result in higher concentrations in the small intestine, where absorption takes place. The impact of gastric pH on the dissolution of posaconazole and its link with systemic exposure was clearly shown where, for instance, a two-fold increase in gastric AUC concentrations would result in a two-fold increase in systemic concentrations of the drug. As there is a major risk that, after intake of the Noxafil® suspension, insufficient therapeutic concentrations of posaconazole are obtained (e.g., depending on the concomitant intake of PPI), formulation scientists at MSD Research Laboratories developed a new formulation that could circumvent this issue (30, 31). In December 2013, the delayed-release tablet formulation of posaconazole was approved and the first studies suggested that this formulation improves the absorption and bioavailability, regardless of gastric pH or food intake (30, 32, 33). Moreover, the daily dose of 400 mg of posaconazole (10 mL of oral suspension) could be reduced to 300 mg for the tablet formulation due to enhanced absorption. Nevertheless, it was not completely clear how this novel formulation was able to generate such a beneficial effect on oral absorption.

Therefore, to unravel the underlying mechanisms that cause the improved intestinal uptake of posaconazole, two clinical GI aspiration studies were performed. In the first study, different suspensions of posaconazole were prepared and administered to healthy subjects in a very small-scale study (*n* = 5) (34). After administration, gastric and intestinal fluids were collected and analyzed for posaconazole concentrations. This study confirmed that posaconazole absorption is limited by its poor solubility in the aspirated intestinal fluids. Being a weak base, however, posaconazole has a higher solubility in the acidic gastric fluids (pH 2). When Posaconazole was dissolved in the stomach and transferred to the small intestine (pH 6), it was observed that intestinal concentrations temporarily exceeded the solubility and that these supersaturated levels of posaconazole were translated into increased absorption. However, this supersaturated state showed a short half-life and posaconazole rapidly precipitated to its limit of solubility, thereby reducing the driving force for intestinal absorption. This study demonstrated for the first time the impact of supersaturated concentrations of a drug in the human intestinal tract on its systemic exposure. For pharmaceutical scientists, the outcome of such studies is highly relevant to guide the development of alternative formulations for posaconazole or similar compounds and to optimize simulation tools. In a follow-up study, the new delayed-release tablet, formulated with the polymer hydroxypropylmethylcellulose acetate succinate (HPMC-AS), was investigated in a clinical aspiration study, including again five healthy subjects (35). The delayed-release properties of this novel tablet should result in a longer maintenance of supersaturated concentrations of posaconazole along the entire GI tract, resulting in a better systemic exposure compared to the posaconazole suspension. In order to profile posaconazole concentrations at a more distal part of the intestinal tract (i.e., jejunum), a customized catheter was designed with a total length of 150 cm. The results of this study clearly confirmed that the new tablet showed a more sustained release of posaconazole resulting in a longer time window of supersaturated concentrations in the jejunum and an increased plasma C_max_ compared to the administration of the suspension. Additional *in vitro* studies demonstrated that HPMC-AS as a precipitation-inhibiting excipient, stabilized the supersaturated state of posaconazole after the delayed release from the tablet (36). Clarifying these mechanisms helps formulation scientists to create a more scientific framework that can assist them in more rational formulation design. Also, the above-mentioned *in vitro* studies clearly demonstrated that HPMC-AS prevents the release of posaconazole in the gastric fluids as this polymer is resistant to an acidic pH. Thus, irrespective of gastric pH (high vs. low), posaconazole's release will be targeted to the intestinal compartment. This work received international recognition from the American Association of Pharmaceutical Scientists (AAPS) [awarded with the “Oral Absorption Focus Group Award” in Orlando, FL (October 2015)].

### Faster and Less Variable Intestinal Absorption of Paracetamol After Intake With Sparkling Water

Besides the impact of GI physiology, the administration conditions may also have a major impact on the systemic exposure of a drug after oral intake (37). One of the “OrBiTo” studies revealed how the intestinal absorption of paracetamol (acetaminophen) was (i) faster and (ii) less variable after intake of a 500 mg immediate-release tablet of Dafalgan® with sparkling water compared to the test condition when paracetamol was taken with still water (38). In a small-scale study, six healthy subjects were recruited and participated in a cross-over study design. In the first arm of the study, a paracetamol tablet was taken with a 330 mL of still water. After intake, gastric fluids were aspirated and analyzed for paracetamol. Simultaneously, blood samples were collected to measure the systemic concentrations of paracetamol and gastric motility was recorded by a high-a resolution manometry (HRM) catheter, which consists of 36 pressure channels to accurately record gastric motility in the human GI tract. The second arm of this study was completely identical as the first arm, except for the intake conditions: the paracetamol tablet was taken with 330 mL of sparkling water instead of still water. It was observed that sparkling water induced transient pressure events along the stomach wall, highly likely stimulating the *in vivo* dissolution rate of the drug. Additional *in vitro* studies demonstrated the faster release of paracetamol in presence of sparkling water compared to still water.

## Concluding Remarks and Future Directions

This 6-year project revealed numerous insights into how GI physiology and formulation strategies have an impact on systemic drug exposure. The optimized biopharmaceutical (*in vitro* and *in silico*) models will allow for a more rational selection of drug and formulation strategies in the pharmaceutical industry, resulting in time- and cost-effective research which is a benefit for pharmaceutical companies and ultimately for patients. These new insights with respect to a better understanding of GI physiology and formulation behavior in the human GI tract (e.g., suspension vs. solid dispersion of posaconazole) have led to an improved knowledgebase for formulation scientists to use in a data-driven, design-led approach when developing new drug products, tailored to the needs of patients. more rationale and pragmatic way of thinking. As the “OrBiTo” project came to an end, new initiatives (e.g., MSCA ITN initiatives such as InPharma, Colotan, UNGAP and AgePOP) recently got launched with the same philosophy: bringing new and better drug products faster on the market for the patient by means of a better understanding of drug product behavior in the human body.

## Author Contributions

BH was invited to write this manuscript in order to contribute to a special issue related to the 10th anniversary of IMI. PA reviewed the entire manuscript and made additional corrections. MM, HL, and BA reviewed the sections with respect to the description of the different work packages and were the main pioneers of this IMI-funded project. A final read was done by BH and MM. All authors contributed to the article and approved the submitted version.

## Conflict of Interest

BH and MM are employed by Pfizer UK. BA is employed by AstraZeneca. The remaining authors declare that the research was conducted in the absence of any commercial or financial relationships that could be construed as a potential conflict of interest.
